# *Legionella pneumophila*: The Paradox of a Highly Sensitive Opportunistic Waterborne Pathogen Able to Persist in the Environment

**DOI:** 10.3389/fmicb.2016.00486

**Published:** 2016-04-08

**Authors:** Jean-Marc Berjeaud, Sylvie Chevalier, Margot Schlusselhuber, Emilie Portier, Clémence Loiseau, Willy Aucher, Olivier Lesouhaitier, Julien Verdon

**Affiliations:** ^1^Laboratoire Ecologie and Biologie des Interactions, UMR CNRS 7267, Université de PoitiersPoitiers, France; ^2^Laboratoire de Microbiologie Signaux et Microenvironnement, EA 4312, Université de RouenEvreux, France; ^3^Laboratoire Aliments Bioprocédés Toxicologie Environnements, EA 4651, Université de CaenCaen, France

**Keywords:** *Legionella pneumophila*, biofilms, amoebae, biocides, natural compounds, antimicrobial peptides, essential oils, biosurfactants

## Abstract

*Legionella pneumophila*, the major causative agent of Legionnaires’ disease, is found in freshwater environments in close association with free-living amoebae and multispecies biofilms, leading to persistence, spread, biocide resistance, and elevated virulence of the bacterium. Indeed, legionellosis outbreaks are mainly due to the ability of this bacterium to colonize and persist in water facilities, despite harsh physical and chemical treatments. However, these treatments are not totally efficient and, after a lag period, *L. pneumophila* may be able to quickly re-colonize these systems. Several natural compounds (biosurfactants, antimicrobial peptides…) with anti-*Legionella* properties have recently been described in the literature, highlighting their specific activities against this pathogen. In this review, we first consider this hallmark of *Legionella* to resist killing, in regard to its biofilm or host-associated life style. Then, we focus more accurately on natural anti-*Legionella* molecules described so far, which could provide new eco-friendly and alternative ways to struggle against this important pathogen in plumbing.

## Introduction

*Legionella pneumophila* is a Gram-negative opportunistic intracellular human pathogen that is responsible for severe pneumonia called Legionnaires’ disease (LD; Fields et al., 2002). The case fatality rate of LD associated with outbreaks is lower than that of sporadic cases, generally around 8–15%, but it can be higher, particularly for hospital-acquired infections, acquired immune-deficiency syndrome (AIDS) patients, transplant patients, and those undergoing aggressive chemotherapy (Dominguez et al., 2009). Among 60 *Legionella* species^1^, *L. pneumophila* is the leading cause of LD and *L. pneumophila* serogroup 1 is associated with almost 85–90% of the cases worldwide (Fields et al., 2002; Yu et al., 2002; Campese et al., 2011; Beaute et al., 2013). Since the first outbreak of pneumonia in 1976 (Fraser et al., 1977; McDade et al., 1977), many LD outbreaks have been linked to various sources of contaminated water in hospitals, hotels, cruise ships, industrial facilities, and family residences (Falkinham et al., 2015). Generally, the economic cost of waterborne diseases including LD is elevated with over $430 million per year in the United States considering only hospitalized patients (Collier et al., 2012). Thus, *L. pneumophila* has a high epidemiological and economical significance, being considered as an opportunistic plumbing pathogen. The bacterium is currently on the United States Environmental Protection Agency (USEPA) candidate contaminant list 4^2^. Transmission to humans occurs after inhalation of contaminated water droplets. *L. pneumophila* reaches the alveolar mucosa and, thanks to its ability to resist phagocytosis, multiplies inside macrophages (Prashar and Terebiznik, 2015). These latter are considered as the primary target of *L. pneumophila* although various data indicate that *L*. *pneumophila* can also invade epithelial cells, in which it can replicate (Cirillo et al., 1994; Mallegol et al., 2014). Its resistance mechanisms to phagocytosis have been thoroughly described and several key steps are highly studied, among which the delivery of effectors into the host cytosol through the Dot/Icm type IV secretion system and the formation of the *Legionella* containing vacuole, which is known as the intracellular replicative niche for the bacterium (Isberg et al., 2009; Hubber and Roy, 2010; Xu and Luo, 2013).

Within freshwater environments, *L. pneumophila* bacteria are ubiquitous organisms, mostly found as parasites of various free-living protozoa such as amoebae, their natural hosts (Steinert et al., 2002). Free-living amoebae are not solely responsible for *L. pneumophila* spreading, but they are also considered as biological shields as they protect intracellular bacteria from adverse conditions or biocide treatments (Loret and Greub, 2010). Thus, amoebae play a key role in the life cycle and pathogenesis of *L. pneumophila*, and its ability to infect human macrophages is thought to be a consequence of prior adaptation to intracellular growth within various primitive eukaryotic hosts such as protozoa (Franco et al., 2009; Al-Quadan et al., 2012). Moreover, upon transfer from natural freshwater habitats into anthropogenic systems, generally at higher temperature than ambient, *L. pneumophila* colonizes existing multispecies biofilms (Rogers et al., 1994). Colonization of these naturally occurring biofilms by *L. pneumophila* can be influenced by several other microorganisms among which protozoa are arguably of particular importance, as they constitute an ecological niche for the pathogen to replicate and to persist (Abdel-Nour et al., 2013). Co-evolution with multiple species of protozoa has resulted in the development of mechanisms that allow *L. pneumophila* to occupy a very broad host range (Abu Kwaik et al., 1998). Biofilms and free-living amoebae are thus considered to serve as main environmental reservoirs for *L. pneumophila* and represent a potential source of drinking water contamination, resulting in a potential health risk for humans (Dupuy et al., 2011; Wingender and Flemming, 2011). Thus, it is of primary importance to find new antibacterial agents to control *L. pneumophila* environmental spread.

This paper presents an overview of the literature regarding the discovery of potential anti-*Legionella* control agents and their mechanisms of action, if known. First, various elements that allow *L. pneumophila* to resist to biocides in its environment are reviewed. Then, the high sensitivity of this bacterium to a diversity of biomolecules that could become of interest in the control of environmental pathogens in water systems is discussed.

## Persistence of *L. pneumophila* in Its Microenvironment

### Resistance of *L. pneumophila* within Biofilms

*Legionella pneumophila* is ubiquitous in natural and anthropogenic water systems, in which it is able to survive for long periods within biofilms (Rogers et al., 1994). Biofilms are defined as complex microbial communities characterized by cells that are attached to a substrate or phase boundary and to each other, and embedded into a matrix of self-produced extracellular polymeric substances (Donlan and Costerton, 2002). Biofilms provide shelter and nutrients, exhibit a remarkable resistance to many stress factors, thus representing an interesting ecological niche for *Legionella* persistence. *L. pneumophila* has also the ability to parasitize protozoa, which commonly graze on biofilm communities (Declerck et al., 2005, 2007). Due to the intracellular lifestyle of *L. pneumophila* within protozoa, it is difficult to tease out whether the resistance of *L. pneumophila* in environmental biofilms is due to the biofilm structure, its association with amoebae or both (Abdel-Nour et al., 2013).

In artificial water systems as in drinking water distribution, *Legionella* growth is detected almost exclusively in biofilms covering the interior of pipe walls, ventilation, and air-conditioning systems, for example (Lau and Ashbolt, 2009). In addition to *Legionella*, these biofilms can become transient or long-term habitats for hygienically relevant microorganisms among which fecal indicator bacteria (*Escherichia coli)*, obligate bacterial pathogens of fecal origin (*Campylobacter* sp.), opportunistic bacteria of environmental origin (*Pseudomonas aeruginosa, Mycobacterium* sp., *Aeromonas* sp.), enteric viruses (adenoviruses, rotaviruses, noroviruses), and parasitic protozoa (*Cryptosporidium parvum*). These organisms can attach to preexisting biofilms, where they become integrated and survive for days to weeks or even longer, depending on the biology and ecology of the organism and the environmental conditions (Blasco et al., 2008; Buse et al., 2014; Richards et al., 2015).

In order to restrain *L. pneumophila* growth, various treatments are used (e.g., physical, thermal, and chemical) in water systems (Jjemba et al., 2015). However, they are not fully efficient, and after a lag period, *L. pneumophila* may be able to quickly re-colonize the system (Thomas et al., 2004; Cooper et al., 2008; Cooper and Hanlon, 2010). Environmental *L. pneumophila* found in biofilms are extremely resilient to treatment with biocides (Kim et al., 2002; Emtiazi et al., 2004; Borella et al., 2005; Saby et al., 2005). When this bacterium is exposed to environmental stresses including biocides and/or found within biofilms, it can enter in a viable but non-culturable state (Giao et al., 2009). The most common biocides used to control waterborne pathogens are generally chlorine derivatives (Walker et al., 1995; Liu et al., 1998; Schwartz et al., 2003; Cervero-Arago et al., 2015). While hyperchlorination of potable water has been shown appropriate for treatment and removal of planktonic cultures of *L. pneumophila*, it remains ineffective against sessile communities (Cooper and Hanlon, 2010; Simoes et al., 2010). Exposure to chlorine at regular intervals has also been shown to facilitate a higher tolerance to disinfectant, thus promoting bacterial resistance (Cooper and Hanlon, 2010). Chlorine dioxide is probably more effective than chlorine because of its superior oxidative power and effect on biofilms (Walker et al., 1995; Hamilton et al., 1996). Chloramine, a powerful chlorine derivative biocide, is a recommended commercial formulation for disinfecting cooling towers. Yet, it has been shown to not completely eradicate *L. pneumophila* from biofilms (Sanli-Yurudu et al., 2007). Recent inquiries into the microbial ecology of distribution systems have shown that pathogen resistance to chlorination is affected by microbial community diversity and interspecies relationships. Multispecies biofilms are generally more resistant to chloramine disinfection than single-species biofilms. One of the reasons may be the presence of nitrifying bacteria leading to depletion of chloramine disinfectant residuals (Berry et al., 2006).

### Resistance of *L. pneumophila* in Association with Its Eukaryotic Hosts

According to the recommendations of the World Health Organization, water disinfection with chlorine has to be performed using a concentration of chlorine between 0.2 and 0.5 mg/l. However, it appears that *Legionella* is recovered if the treatment is not continuous. The persistence of *L. pneumophila* is due, at least partly, to its intra-amoeba lifestyle (Steinert et al., 1998; Hilbi et al., 2011) since these protozoa act as biological shields, protecting bacteria from biocides (Loret and Greub, 2010). Amoeba-grown *L. pneumophila* are thus more resistant than planktonic cells to chemical disinfectants, biocides (Barker et al., 1992; Dupuy et al., 2011), and antibiotics (Barker et al., 1995). It has been indeed reported that intracellular *L. pneumophila* are released from amoebae within vesicles containing several hundreds of resistant bacteria to biocides such as the isothiazolone-derivative minimum bactericidal concentration (MBC215; a mixture of 5-chloro-2-methyl-4-isothiazolin-3-one and 2-methyl-4-isothiazolin) and the quaternary ammonium compound poly(oxyethylene) (dimethylimino) ethylene (dimethylimino) ethylene dichloride (Berk et al., 1998). In addition, bacteria released through these vesicles were viable up to 6 months (Bouyer et al., 2007). In the same way, it has been demonstrated that amoebae promote resuscitation of viable but non-culturable *Legionella*, enhancing in parallel their resistance to sodium hypochlorite (Garcia et al., 2007). The level of *Legionella* resistance into amoebae is also dependent of the disinfectant used. For example, monochloramine displays the same efficiency against planktonic bacteria in the presence or not of amoebae whereas chlorine and chlorine dioxide are less active against *Legionella* co-cultured with amoebae (Dupuy et al., 2011). The understanding, at the molecular level, of the intra-protozoa acquired *Legionella* resistance (Garduno et al., 2002; Koubar et al., 2011) is obviously an important information in order to develop strategies to limit or eradicate this phenomenon. Interestingly, it was also shown that *L. pneumophila* resistance against chlorine acquired into amoebae is host-dependent (Chang et al., 2009), suggesting the involvement of specific molecular mechanisms currently unknown.

In conclusion, the use of biocides such as chlorine or chloramine to disinfect water appears to only limit the development of *L. pneumophila* without being able to eradicate this pathogen (Wang et al., 2012). More critically, intracellularly grown *L. pneumophila* become more resistant when exposed to biocides (Cervero-Arago et al., 2015), suggesting that it is necessary to control, in water, both bacterial pathogens and their natural hosts like amoebae. In this way, studies of the direct impact of biocides on amoebae, alone or infected with a pathogen, would be very helpful (Srikanth and Berk, 1994; Mogoa et al., 2010, 2011; Fouque et al., 2015). Noticeably, infected amoebae were shown to become more pathogenic than uninfected amoebae (Brieland et al., 1997), suggesting that the two partners (i.e., amoebae and *L. pneumophila*) enhance synergistically their pathogenesis. Therefore, studies dealing with treatments against Legionella should be performed on bacteria associated with their natural host in order to (i) determine how the bacteria are protected during their intracellular life cycle, (ii) if the passage inside host cells modifies the Legionella resistance after its escape, and (iii) how the host responds to treatments (Thomas et al., 2004; Donlan et al., 2005; Dupuy et al., 2011).

## Natural Biocides: An Alternative Way to Control *L. pneumophila* Spread in the Environment?

For a few years now, some studies have highlighted natural compounds with anti-*Legionella* properties. Why such an interest? Probably because (i) *L. pneumophila* is a waterborne bacterium ubiquitously found in freshwater environments, (ii) LD is a severe and sometimes fatal multisystem illness involving atypical pneumonia, (iii) the development of man-made water systems such as air-conditioners and cooling towers has expanded the environmental niche of *L. pneumophila* in association with amoebae, (iv) emerging pathogens such as *L. pneumophila* or spore-forming bacteria such as *Bacillus* are able to resist to currently used water disinfection procedures, and (v) more efforts are needed to control disinfection by-products and minimize people exposure to potentially hazardous chemicals (Trihalomethanes, Haloacetic acids…) while maintaining adequate disinfection and control of targeted pathogens while respecting the environment. Subsequently, we review in this chapter recent advances in finding natural compounds exhibiting direct or indirect anti-*Legionella* activity and discuss, if known, their mode of action.

### Proteins

**FIGURE 1:**
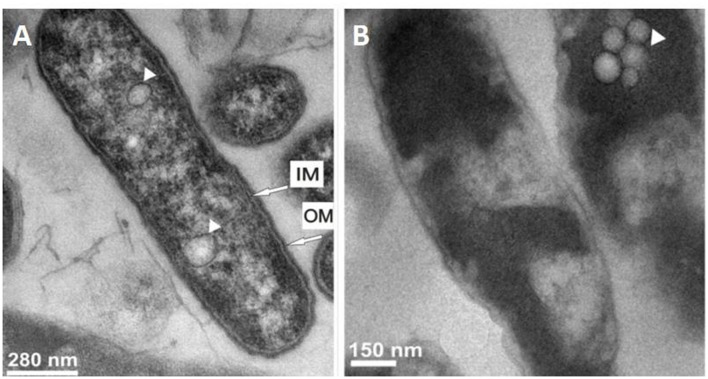
**Ultrastructural changes in *Legionella dumoffii* cells after treatment with *Galleria mellonella* apolipophorin III.** Transmission electron micrographs of **(A)** untreated bacteria and **(B)** treated bacteria with 0.4 mg/ml ApoLp-III. The presence of vacuoles is indicated by arrowhead. IM, Inner membrane; OM, Outer membrane (Source: Palusinska-Szysz et al., 2012).

To date, only two proteins have been found to be directly active against *Legionella* cells: the greater wax moth *Galleria mellonella* apolipophorin III (ApoLp-III) and the human lactoferrin. The ApoLp-III protein family is composed of low-molecular weight apolipoproteins (161–166 amino acid residues) characterized by a globular amphipathic α-helix bundle conformation (Weers and Ryan, 2006). ApoLp-III has been shown to be an important component of hemolymph of numerous insect species of *Orthoptera, Leptidoptera, Coleoptera*, and *Hemiptera* genus. In addition, ApoLp-III is involved in lipid transport and immunity. Recently, the protein was recovered by methanol extraction, purified and evaluated against three *Legionella* species: *L. dumoffii, L. pneumophila*, and *L. gormanii* (Palusinska-Szysz et al., 2012; Chmiel et al., 2014; Zdybicka-Barabas et al., 2014). Antimicrobial assays demonstrated a difference in susceptibility among *Legionella* species. An 1-hincubation time of cells with 0.1 mg/ml of protein induced a moderate mortality rate of 55% for *L. pneumophila* vs. 40% for *L. gormanii*. The highest protein concentration tested in the studies, 0.4 mg/ml for *L. dumoffii*, 1.6 mg/ml for *L. pneumophila*, and 0.2 mg/ml for *L. gormanii* decreased the survival rate of 30, 100, and 50%, respectively. The effect of the protein on *L. dumoffii* was also investigated by transmission electron microscopy (Palusinska-Szysz et al., 2012). This study highlighted cell wall damages and strong intracellular alterations, such as increased vacuolization and condensation in the cytoplasm (**Figure 1**). Interestingly, cell envelope damages appeared greater for bacteria cultured on medium with choline supplementation (Palusinska-Szysz et al., 2012). Consistently, the sensitivity toward ApoLp-III of this species was threefold increased when cells were grown in the presence of choline. Extracellular choline is known to be used by some *Legionella* species for the synthesis of phosphatidylcholine (PC) that are phospholipids commonly found as component of eukaryotic membranes while only encountered in the envelope of about 15% of bacteria (Martinez-Morales et al., 2003; Geiger et al., 2013; Sohlenkamp and Geiger, 2016). Based on these observations as well as atomic force microscopy (AFM), Fourier transform infrared spectroscopy (FTIR), and lipopolysaccharide (LPS) binding studies, the authors assumed that ApoLp-III interacts with lipid components of *Legionella* cell membrane (Zdybicka-Barabas et al., 2014). Indeed, the protein most probably interacts with phospholipids (especially PC) of *L. dumoffii* while the anti-*L. pneumophila* effect is rather driven by interaction with LPS and other lipid components of its membrane. ApoLp-III shares homology with the 22-kDa N-terminal domain of the human apolipoprotein E (ApoE). This 37-kDa apolipoprotein has similar roles as ApoLp-III including lipid transport, host immunity as well as immunomodulatory properties. As for its insect homolog, SDS-PAGE and FTIR analysis revealed that ApoE strongly interacts with LPS of *L. pneumophila* outer membrane. However, although AFM analysis demonstrated alterations in the cell surface topography and properties, 0.8 mg/ml of protein did not reduce viability of *L. pneumophila* cells after an 1-h treatment (Palusinska-Szysz et al., 2015).

Lactoferrin is a glycoprotein of the transferrin family found at high levels in milk. The molecule, thanks to its ferric ions binding capacity, presents multiple biological functions. Indeed, lactoferrin is known to interact with the molecular and cellular components of hosts and pathogens (Siqueiros-Cendon et al., 2014). Bortner et al. (1986, 1989) studied the anti-*Legionella* activity of the human lactoferrin under both its iron-free and iron-saturated states. The study reveals a difference in terms of activity between the two states of the protein. Indeed, 0.09 mg/ml of apolactoferrin (the iron-free state) was able to kill 99.99% of exponentially grown *Legionella* cells after 2 h of incubation, while the iron-saturated form was unable to reduce viability of the bacteria in the same conditions (Bortner et al., 1986). The activity of apolactoferrin was abrogated at temperatures below 22°C and by the addition of MgCl_2_, CaCl_2_, or Mg(NO_3_)_2_ but not NaCl (Bortner et al., 1986, 1989). The physiological state of *Legionella* also played a role in the sensitivity toward apolactoferrin since stationary phase cells became more resistant to the protein. The mechanism by which apolactoferrin kills *Legionella* is currently unknown. Previously, the bactericidal activity of lactoferrin against other Gram-negative bacteria was shown to be mediated through the binding of the protein to receptors on the bacterial surface inducing cell-death due to a disruption in the cell wall (Jenssen and Hancock, 2009). The bactericidal activity against Gram-positive bacteria is mediated by electrostatic interactions between the positively charged protein and the bacterial membrane leading to its permeabilization (Jenssen and Hancock, 2009).

### Protein-Derived Peptides

Regarding protein-derived peptides, two synthetic fragments of protein were shown to have anti-*Legionella* activity. The first one, C18G (ALYKKLLKKLLKSAKKLG; 2043 Da), is based on the antimicrobial peptide C13 corresponding to the last 13 amino acids of the carboxyl terminus of human platelet factor IV. The peptide was designed to improve its antibacterial potency by increasing the length of C13 and substituting a negative charge with a positive charge (Darveau et al., 1992). The activity of the synthetic amphipathic α-helical cationic peptide has been evaluated against *L. pneumophila* (Robey et al., 2001). Minimal bactericidal concentration was determined on logarithmic-phase bacteria as 32 to 128 μg/ml, depending on Mg^2+^ concentration. Deletion of *rcp* gene, encoding a protein with homology to the lipid A palmitoyltransferase PagP of *Salmonella* serovar Typhimurium and *E. coli*, led to a slight increase in susceptibility of the bacteria to the peptide indicating that this gene is involved in the resistance to cationic antimicrobial peptides by a Mg^2+^ mediated pathway. This latter is linked to the addition of palmitate on LPS, leading to a decrease of membrane fluidity thus preventing peptide insertion (Guo et al., 1998).

When compared to C18G, another synthetic protein fragment named NK-2 demonstrated its efficacy in *Legionella* killing. NK-2 (KILRGVCKKIMRTFLRRISKDILTGKK; 3203 Da) was designed as the partial sequence of the porcine lymphatic effector protein NK-lysin corresponding to the core region of the protein (residues 39–65) (Leippe, 1995). Various studies highlighted the very high potency of the peptide in the killing of cancer cells and various pathogens including Gram-negative and Gram-positive bacteria, the yeast *Candida albicans* as well as the intracellular parasites *Trypanosoma cruzi* and *Plasmodium falciparum* (Andra and Leippe, 1999; Jacobs et al., 2003; Schroder-Borm et al., 2003; Gelhaus et al., 2008; Jena et al., 2011). Interestingly, NK-2 appears to be non-hemolytic with no cytotoxicity toward normal mammalian cells such as keratinocytes, lymphocytes, macrophages, and glioblastoma cells at bactericidal concentrations (Andra and Leippe, 1999; Jacobs et al., 2003; Schroder-Borm et al., 2003; Jena et al., 2011). Recently, the knowledge about the potency of the synthetic peptide was extended to *Legionella*. The minimal bactericidal concentration was determined on exponentially grown *L. pneumophila* (Schlusselhuber et al., 2015). The study revealed that a concentration of 1.6 μM was able to kill the bacteria. NK-2 is a cationic peptide that adopts an amphipathic alpha helical secondary structure upon membrane interaction. A previous study revealed the possible mode of action of the peptide (Willumeit et al., 2005). Indeed, NK-2 was shown to bind and permeabilize membranes containing negatively charged phosphatidylglycerol (PG; found in the cytoplasmic membranes of bacteria) whereas no effects were observed with pure zwitterionic PC model membranes (as a mimetic for human cell membranes). Regarding phosphatidylethanolamine (PE) model membranes (a major phospholipid component of bacterial cell membranes), NK-2 binds and slightly inserts into the model membrane. The direct interaction with lipids leads to an increase of the membrane stiffness, thus favoring the formation of inverted lipid structures promoting the intrinsic negative membrane curvature. Enhancement of this effect results in membrane tension and disruption (Willumeit et al., 2005).

### Antimicrobial Peptides (AMPs)

Historically, the first antimicrobial peptides tested against *L. pneumophila* were some apidaecin-type peptides, consisting in proline-rich molecules isolated from various hymenopteran insects (Casteels et al., 1994). Interestingly, in the same study the most active anti-*Legionella* peptide tested was Cecropin P1, a 31 amino acids long peptide isolated from the pig small intestine (Lee et al., 1989). However, the antimicrobial activities of these peptides were only estimated from the diameter of the inhibition zones observed on agar plates.

**FIGURE 2:**
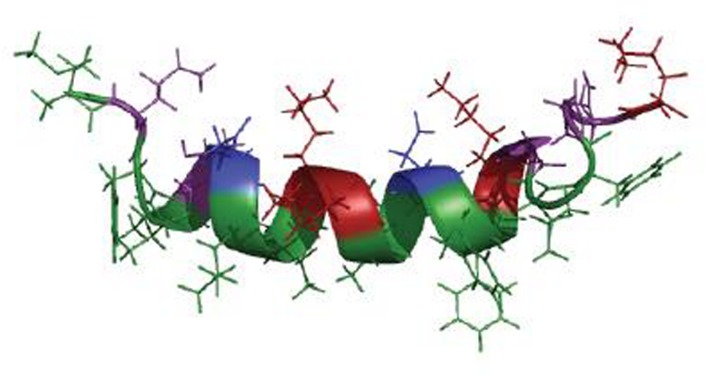
**Structural model of warnericin RK in a membrane-like environment.** Green: hydrophobic residues; Purple: hydrophilic and neutral residues; Blue: negatively charged residues; Red: positively charged residues (Source: Adapted from Verdon et al., 2009a).

The first anti-*Legionella* peptides, produced by bacteria, were purified and characterized from the culture supernatant of a *Staphylococcus warneri* strain. This strain, *S. warneri* RK, was first detected as a contaminant colony on a *L. pneumophila* culture surrounded by a characteristic inhibition zone (Hechard et al., 2005). This activity was assigned to a molecule secreted by *S. warneri* RK. This molecule displayed a high heat-stability and its activity was lost after protease treatments, indicating that it might be an antimicrobial peptide. Finally, three anti-*Legionella* peptides produced by *S. warneri* RK were characterized (Verdon et al., 2008). One peptide, warnericin RK, is original, while the two others are delta-lysin I and delta-lysin II, encoded by genes previously described (Tegmark et al., 1998). They are close to *S. aureus* delta-hemolysin which was known for its action on red blood cells and was deemed to be devoid of antibacterial activity (Verdon et al., 2009b). The *S. warneri* peptides share similar biochemical characteristics as they are short (22 amino acids), cationic and highly hydrophobic. They display the same antibacterial spectrum, which is almost restricted to the *Legionella* genus. However, the amino acids sequence alignment (**Table 1**) shows that no high similarity exists between the three peptides even if they were predicted to adopt an α-helical structure (Verdon et al., 2008). This structure was further confirmed for warnericin RK by circular dichroism (CD) and nuclear magnetic resonance (NMR) spectroscopy analyses (Verdon et al., 2009a). CD spectroscopy showed that the peptide did not have a defined secondary structure in aqueous solution. However, in a membrane-like environment that is mimicked by the addition of dimyristoylphosphatidylcholine vesicles or 8% trifluoroethanol (TFE), a defined α-helical secondary structure was formed. From the 2D-NMR analysis, performed in 8% TFE, NOESY spectra revealed a well-defined α-helix extending from residue 4 to residue 16 (**Figure 2**). This central part of the peptide forms a nearly perfect amphiphilic helix, which is also observed in the *S. aureus* delta-hemolysin (Lee et al., 1987).

**Table 1:** Anti-*Legionella* antimicrobial peptides (AMPs) produced by Staphylococci (Adapted from Marchand et al., 2011).

	Peptide	Producing bacteria	Amino acids sequence (N_ter_–Ct_ter_)	MIC (μM)
Group 1	Warnericin RK	*S. warneri*	MQFITDLIKKAVDFFKGLFGNK	0.3
	δ-Lysin I*	*S. warneri*	MAADIISTIGDLVKLIINTVKKFQK	1.08
	δ-Lysin II	*S. warneri*	MTADIISTIGDFVKWILDTVKKFTK	0.54
	δ-Hemolysin	*S. aureus*	MAQDIISTIGDLVKWIIDTVNKFTKK	1.05
	Ggi I	*S. haemolyticus*	MQKLAEAIAAAVSAGQDKDWGKMGTSIVGIVENGITVLGKIFGF	4.15
	SLUSH C	*S. lugdunensis*	MDGIFEAISKAVQAGLDKDWATMGTSIAEALAKGVDFIIGLFH	5.16
	SLUSH A	*S. lugdunensis*	MSGIVDAITKAVQAGLDKDWATMATSIADAIAKGVDFIAGFFN	11.28
Group 2	PSMα	*S. epidermidis*	MADVIAKIVEIVKGLIDQFTQK	0.63
	δ-Hemolysin	*S. epidermidis*	MMAADIISTIGDLVKWIIDTVNKFKK	1.59
	PSMβ	*S. epidermidis*	MSKLAEAIANTVKAAQDQDWTKLGTSIVDIVESGVSVLGKIFGF	2.69
	H2U*	*S. cohnii*	MDFIIDIIKKIVGLFTGK	3.04
	Ggi II	*S. haemolyticus*	MEKIANAVKSAIEAGQNQDWTKLGTSILDIVSNGVTELSKIFGF	13.23
	Haemo 3	*S. haemolyticus*	n.d.	1.38

Minimum inhibitory concentrations were determined against *L. pneumophila* Lens and for the formylated forms of the peptides except for the peptides indicated by *.

Several anti-*Legionella* peptides have been found so far, mainly in bacteria belonging to the *Staphylococcus* genus (Marchand et al., 2011). Indeed, nine strains representing nine different species of staphylococci were found to secrete anti-*Legionella* compounds. All the purified compounds (**Table 1**), except one (Haemo 3 from *S. haemolyticus*), corresponded to previously described hemolytic peptides and were not known for their anti-*Legionella* activity. It should be noted that, beside the non-substituted peptides, the N-formylated forms (N-formylmethionine) of these compounds have been isolated and are active against *Legionella* (Verdon et al., 2008; Marchand et al., 2011). Moreover, the formylated forms of warnericin RK, δ-hemolysin II from *S. warneri*, and PSMα from *S. epidermidis* showed a higher inhibitory activity (MIC < 0.6 μM) than the corresponding non-substituted forms (MIC > 1.1 μM) (Marchand et al., 2011). These three peptides were found active against all the *Legionella* tested, corresponding to 6 *L. pneumophila* strains belonging to 4 different serogroups (1, 3, 5, and 6) and 6 non-*pneumophila* species. They appear to be very specific of the *Legionella* genus. Nevertheless, all the 12 anti-*Legionella* peptides described to date (Verdon et al., 2008; Marchand et al., 2011) display hemolytic activity.

On the basis of their antimicrobial [minimum inhibitory concentration (MIC), minimum permeabilization concentration, decrease of bacterial cultivability] and hemolytic activities, the purified peptides were separated into two groups (**Table 1**). The first group, including warnericin RK, corresponds to highly hemolytic and bactericidal peptides. The peptides of the second group, including PSMα from *Staphylococcus epidermidis*, are bacteriostatic and poorly hemolytic. Thus, a structure/activity relationships study was performed on the archetypes of each group of anti-*Legionella* peptides, warnericin RK and PSMα, in order to determine key amino acids (Marchand et al., 2015). Firstly, it was shown that the predicted helical wheel projections of these two peptides, assuming that the whole sequences were in an ideal α-helical structure, appeared similar when one of the sequence was reversed. Consequently, the authors designed a library of variants by replacing selected amino acids from one sequence by the corresponding of the reverse sequence of the other. Comparison of the anti-*Legionella* and hemolytic activities of these variants with these of the parent peptides succeeded in determining specific amino acid residues in warnericin RK and PSMα sequences that are critical. Surprisingly, the residue in the 14th position in both sequences (Phenylalanine for warnericin RK and Glycine for PSMα) appeared crucial for hemolytic activity but not for antibacterial activity (**Table 1**). However, as expected, the authors showed that the antibacterial activity of such peptides was correlated with their global positive charge.

Only the mode of action of warnericin RK has been studied in detail so far. A concentration of this peptide equals or superior to 3.12 μM was shown to fully suppress the growth ability of *L. pneumophila* (Verdon et al., 2009a). By using planar lipid bilayer studies and osmotic protection experiments, it was suggested that warnericin RK is membrane active. More precisely, results indicated that warnericin RK forms large channels of various sizes in erythrocytes as well as in model lipid membranes (Verdon et al., 2009a). This means that warnericin RK is likely to have a detergent-like mode of action, as detailed for several others AMPs (Bechinger and Lohner, 2006; Haney et al., 2009). The peptides indeed self-associate and transiently destabilize the membrane. At higher concentrations, this destabilization could lead to cell lysis. Furthermore, it was demonstrated that *Legionella* is particularly sensitive to detergents (by 10- to 1000-fold) in comparison to other tested bacteria (Verdon et al., 2009a), which is fully consistent with a putative detergent-like mode of action for warnericin RK.

The specific sensitivity of *Legionella* to warnericin RK, and probably to detergents, seems to be related to the lipid composition of its membrane and not to the presence of a dedicated proteinaceous receptor. This was confirmed by Verdon et al. (2011) who tried unsuccessfully to obtain a *Legionella* mutant resistant to warnericin RK by screening a collection of mutants obtained by transposition mutagenesis. However, in the same study, the authors isolated an adapted strain which was able to grow at a concentration 33-fold higher than the MIC of the wild type strain. Therefore, the comparison of the fatty acids content of the wild type and adapted strains cell membranes indicated that the increase in branched-chain fatty acids and the decrease in fatty acid chain length in cell membranes were correlated with an increase in resistance to warnericin RK. Therefore, the fatty acids profile seems to play a critical role in the sensitivity of *L. pneumophila* to warnericin RK (Verdon et al., 2011). The other characteristic of the lipid composition of the *Legionella* membrane consists in its high level (30%) of PC which is mainly considered as an eukaryotic phospholipid (Hindahl and Iglewski, 1984; Conover et al., 2008).

**FIGURE 3:**
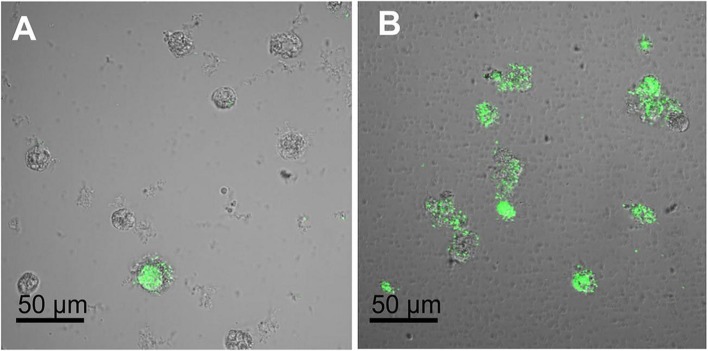
**Activity of Ci-MAM-A24 against intra-amoebic *L. pneumophila* observed by confocal microscopy.** *A. castellanii* cells infected with GFP expressing *L. pneumophila* Lens were incubated 6 h post-infection with **(A)** 12.5 μM of Ci-MAM-A24 or **(B)** peptide solvent during 42 h (48 h post-infection) (Source: Schlusselhuber et al., 2015).

Anti-*Legionella* activity of AMPs from a non-prokaryotic source was also described in the literature. To date only three peptides, among which two were derived from natural AMPs of the marine organism *Ciona intestinalis* and one was purified from the greater wax moth *Galleria mellonella*, have been studied. Ci-MAM-A and Ci-PAP-A are naturally present in the ascidian tunic as well as in granulocytes of inflamed tissues of *C. intestinalis*, thus constituting a chemical protection to microbial invasion for this organism (Fedders and Leippe, 2008; Di Bella et al., 2011). The authors assumed, based on the knowledge about the processing of AMPs precursors, that sequences of Ci-MAM-A and Ci-PAP-A may represent the prepropeptides of two mature cationic peptides. Therefore, two synthetic peptides, named Ci-PAP-A22 and Ci-MAM-A24, were designed, and represent the cationic amphipathic regions of these two precursors (Fedders and Leippe, 2008; Fedders et al., 2008). Both share a similar size (22–24 amino acid residues) and the propensity to adopt an amphipathic alpha-helical structure. Synthetic peptides were shown to be microbicidal at low micromolar concentrations (below 12.5 μM) against various Gram-positive and Gram-negative bacteria as well as the fungus *Candida albicans* (Fedders and Leippe, 2008; Fedders et al., 2008). Moreover, Ci-MAM-A24 is extraordinarily salt tolerant and was also found to be remarkably effective against mycobacteria as well as multi-resistant clinically important aerobic and anaerobic strains (Fedders et al., 2008; Jena et al., 2011). Recently the anti-*Legionella* and the anti-*Acanthamoebae* activities of these two highly potent peptides were evaluated (Schlusselhuber et al., 2015). The EC_50_ (concentration that kills 50% of *Legionella* planctonic cells) was very low for both peptides, below 0.5 μM. However, when considering the minimal bactericidal concentration, Ci-MAM-A24 was found to be much more effective against *Legionella* cells (1.6 μM) compared to Ci-PAP-A22 (25 μM). The anti-*Acanthamoeaba* activity of peptides was also determined. The highest concentration tested (25 μM) led to 85% permeabilization of cells by Ci-MAM-A24, but showed no significant effect below this concentration. In comparison, Ci-PAP-A22 did not induce significant permeabilization at these concentrations. Interestingly, the most effective peptide, Ci-MAM-A24, was also found to reduce the intra-amoebae *Legionella* cell number at a non-toxic concentration for the host cell (12.5 μM), as illustrated in **Figure 3**. In the frame of elaborating anti-*Legionella* surfaces, the peptide was then immobilized on gold surfaces to assess its antimicrobial activity. The study revealed that the potent bactericidal activity of the peptide was conserved after its immobilization (Schlusselhuber et al., 2015). CD measurements clearly showed that Ci-PAP-A22 and Ci-MAM-A24 undergo a distinct conformational change upon interaction with some liposomal membranes (Fedders et al., 2008). After mixing with anionic phospholipids (especially PG, L-α-phosphatidyl-DL-glycerol and phosphatidylserine (PS), L-α-phosphatidylserine), both peptides adopted an α-helical structure. Indeed, the CD spectra exhibited the typical shape of a linear peptide in the absence of liposomes, while the typical minima at 222 and 208 nm appeared after mixing with PG or PS. Moreover, with those lipids, peptides adopted a parallel orientation to the membrane surface. The killing activity of these peptides was found to be due to membrane permeabilization. However, a minimalistic system using the depolarization of liposomes revealed a weak pore-forming activity. These data suggested that Ci-MAM-A24 and Ci-PAP-A22 act more likely via a carpet or toroidal-type mechanism, leading to transient pore formation (Fedders et al., 2008).

The *Galleria* defensin, a 43 aminoacids long peptide (Cytrynska et al., 2007), isolated from greater wax moth *Galleria mellonella*, was showed to be active against *Legionella dumoffii* (Palusinska-Szysz et al., 2012). Interestingly, it was shown that the bacteria grown on choline supplemented medium were more sensitive to the peptide than those grown on non-supplemented medium. Like other *Legionella* species (*L. pneumophila, L. bozemanae, L. lytica*), *L. dumoffii* can use extracellular choline for the synthesis of PC. As a consequence, it could be postulated that there is a direct relationship between the level of PC in the *Legionella* membrane and its sensitivity to the *Galleria* defensin. Moreover, the lytic activity of δ-hemolysin from *S. aureus* toward di-palmitoyl-PC vesicles was described (Laabei et al., 2014). This hemolytic peptide was also shown to display an antimicrobial activity restricted to the *Legionella* genus (Marchand et al., 2011). Taken together, these data suggest that the peculiar sensitivity of bacteria from the *Legionella* genus to specific AMPs could be related to the high content of PC in its membrane. However, it was shown that, contrary to *L. dumoffii*, choline supplementation did not induce higher sensitivity of *L. pneumophila* to ApoLp-III (Zdybicka-Barabas et al., 2014), and the authors suggested that the sensitivity of the bacteria was related to the interaction of the antimicrobial protein and the LPS.

## Essential Oils (EOs)

Essential oils are aromatic oily liquids obtained from plant material such as flowers, buds, seeds, leaves, twigs, bark, herbs fruits, or roots, and are mainly composed of a mixture of terpenoïds and aromatic compounds. Among terpenes, monoterpenes, diterpenes, and sesquiterpenes are the most currently found (Dorman and Deans, 2000; Bakkali et al., 2008; Mkaddem et al., 2009). EOs are classified according to the chemical nature of their main active components (Burt, 2004; Bakkali et al., 2008). EOs are well known to possess a wide spectrum of antagonistic activities like antibacterial (Burt, 2004; Solorzano-Santos and Miranda-Novales, 2012; Silva et al., 2013; Seow et al., 2014), antiviral (Reichling et al., 2005; Saddi et al., 2007), antifungal (*Penicillium expansum*, *Botrytis cinerea*, and *Candida oleophila*) (Mari et al., 2003), antitoxigenic (Mycotoxin) (Juglal et al., 2002), antiparasitic (Tariku et al., 2011; Rodrigues et al., 2013) or acaricidal (Neves and da Camara, 2011), and insecticidal activities (*Drosophila*) (Karpouhtsis et al., 1998).

Investigations were performed by Chang et al. (2008b) to assess the antibacterial activity of EOs against *L. pneumophila*. Indeed, the authors determined the anti-*L. pneumophila* activity of EOs extracted from *Cinnamomun osmophloeum* leaves and from different tissues of *Cryptomeria japonica.* Among the ten kinds of EOs tested, those extracted from *C. osmophloeum* leaves exhibited a stronger anti-*Legionella* activity than those extracted from *C. japonica*. More precisely, the highest bactericidal effect was obtained with the *C. osmophloeum* leaf EO of cinnamaldehyde type (characterized by its major constituent, cinnamaldehyde, accounting for 91.3% of EO) (**Table 2**). The great bioactivity of cinnamon oil appears to be a promising candidate for controlling *Legionella* growth in recreational spring water and possibly other environments generally at basic pH, i.e., cooling towers (Chang et al., 2008a).

**Table 2:** Major components and minimum bactericidal concentration (MBC) or MIC of EOs that exhibit anti-*Legionella pneumophila* properties.

Common name of EO	Latin name of plant source	Major components	Approximate concentration (%)	*MBC_100_ (MIC)	Reference
Cinnamon	*Cinnamomum osmophloeum*	Trans-Cinnamaldehyde Benzenpropanal 4-allylanisole	91.32 3.18 1.42	1000 μg/ml	Chang et al., 2008b
Tea tree	*Melaleuca alternifolia*	Terpinen-4-ol 1,8-Cineole	42.35 3.57	0.5% v/v	Mondello et al., 2009
Juniper	*Juniperus phoenicea*	Isoborneol 1S-α-Pinene	20.91 18.30	(0.03 mg/ml)	Chaftar et al., 2015b
Thyme	*Thymus vulgaris*	Carvacrol	88.50	(0.07 mg/ml)	Chaftar et al., 2015b

*MBC_*100*_: The minimum bactericidal concentration of EO that inactivated at least 99.9% of the bacteria. MICs values are indicated in brackets.

In Mondello et al. (2009), a study was conducted to determine the antimicrobial activity of *Melaleuca alternifolia cheel* (tea tree) oil (TTO) against 22 strains of *L. pneumophila* of various serogroups and sources of isolation. Results showed that *L. pneumophila*, quite irrespectively of serogroups and sources of isolation, is highly sensitive to TTO, with MICs ranging from 0.125 to 0.5% v/v, and a minimum bactericidal concentration (MBC_100_) at 0.5% v/v (**Table 2**). Therefore, TTO could be used as an anti-*Legionella* disinfectant for the control of water system contamination, specifically in spa, small waterlines, or in respiratory medical devices.

Recently, the effects of *Citrus* EOs vapors were tested on different strains of *Legionella* in water and soil systems (Laird et al., 2014). Among all the tested strains, an antagonistic effect was observed on *L. pneumophila*, *L. longbeachae*, *L. bozemanii*, and on *intra-amoebae* cultured *L. pneumophila* with an acute susceptibility for *L. pneumophila* in water. Different systems of vapors production (passive and active sintering of the vapor) were tested. EOs vapors components were identified (linalool, β-pinene, and citral) and their antimicrobial efficacy was determined. There was up to a 5-log cells/ml reduction in *Legionella* sp. in soil after exposure to the citrus EO vapors (15 mg/l air). Moreover, data showed that sintering the vapor through water increased the presence of antimicrobial components, including an increase of linalool (57.17 mg/l) compared to the passive system (35.43 mg/l). Thus, the appropriate method for delivering *Citrus* EO vapor may go some way in controlling *Legionella* spp. from environmental sources (Laird et al., 2014).

More recently, results obtained by Chaftar et al., 2015a,b), have highlighted the anti-*Legionella* activity of essential oils extracted from Tunisian plants. Oils extracted from *Juniperus phoenicea* and *Thymus vulgaris* exhibited the highest anti-*L. pneumophila* activity, with MICs lower than 0.03 mg/ml and lower than 0.07 mg/ml, respectively. *J. phoenicea* oil is mainly composed of isoborneol (20.91%), (1S) α-pinene (18.30%), β-phellandrene (8.08%), α-campholenal (7.91%), and α- phellandrene (7.58%). Concerning the *T. vulgaris* oil, carvacrol (88.50%) and *p*-cymene (7.86%) were the major components (**Table 2**).

**FIGURE 4:**
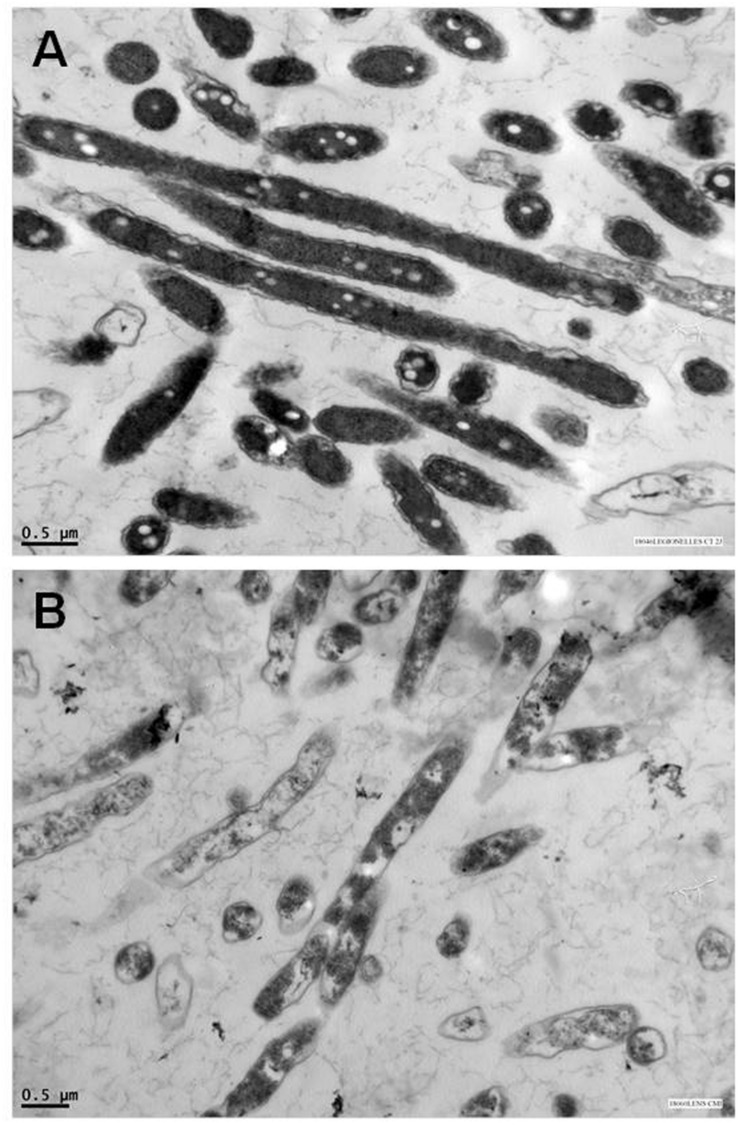
**Anti-*Legionella* activity of *Thymus vulgaris* EO observed by transmission electron microscopy.** Micrographs of **(A)** untreated control cells of *L. pneumophila* Lens strain and **(B)** treated *L. pneumophila* Lens with 70 μg/ml *Thymus vulgaris* EO (Source: Chaftar et al., 2015b).

In regard to EOs composition and relative abundance variabilities, their antibacterial activity could not be link to one specific mechanism as cells possess several targets (Carson et al., 2002). Indeed, EOs can act by degrading the cell wall (Helander et al., 1998), inducing damages to the cytoplasmic membrane (Ultee et al., 2002) and to membrane proteins (Ultee et al., 1999), causing the leakage of cell contents (Lambert et al., 2001), coagulating the cytoplasm (Gustafson et al., 1998) and depleting the proton motive force (Ultee and Smid, 2001). To date, only Chaftar et al. (2015b) have investigated EOs action against *L. pneumophila*. Indeed, scanning electron microscopy analysis highlighted morphological alterations of bacteria when treated with *T. vulgaris* EO as cells appeared shorter, flattened, and expanded compared to the untreated ones. Transmission electron microscopy experiments indicated that treated *L. pneumophila* cells were less homogeneous and electron-dense than the untreated control, suggesting a loss of membrane integrity (**Figure 4**). Authors hypothesized that carvacrol, which constituted 88.5% of the present oil, might destabilize the cytoplasmic membrane and acts as a proton exchanger, as it was shown to alter cell membranes fluidity and permeability, due to its lipophilic properties. However, precise mechanistic data are needed to validate this hypothesis.

### Biosurfactants

Biosurfactants are a structurally diverse group of surface-active molecules produced by various microorganisms: bacteria, yeasts or fungi (Pacwa-Plociniczak et al., 2011). Indeed, they are biological amphiphiles composed of a hydrophobic moiety containing saturated, unsaturated, and/or hydroxylated fatty acids or fatty alcohols, and a hydrophilic moiety consisting of mono-, oligo-, or polysaccharides, peptides or proteins (Lang, 2002). Mostly depending on their chemical composition and their molecular weight, biosurfactants are commonly classified as low (including glycolipids, phospholipids, and lipopeptides) and high-molecular weight (polysaccharides, proteins, lipoproteins, and LPS) compounds (Pacwa-Plociniczak et al., 2011). They play critical roles in several biological processes such as the metabolism of hydrophobic substrates, biofilms development and maintenance, biofilms disruption and/or prevention, bacterial motility, host–microbe interactions, stimulation of the induced systemic resistance phenomenon or by acting as natural biocide. Therefore, they are considered of great interest for several biotechnological, biocontrol, and therapeutic applications (Ongena and Jacques, 2008; Sen, 2010; Gudina et al., 2013). While biosurfactants were reported to exhibit lytic and growth-inhibitory activities against a broad range of microorganisms, including viruses, mycoplasmas, bacteria, fungi, and oomycetes, only one study has reported anti-*Legionella* activity so far (Loiseau et al., 2015). Therefore, several surfactin isoforms were shown to display an antibacterial spectrum almost restricted to the *Legionella* genus (MICs range 1–4 μg/ml), and also to exhibit a weak activity toward the amoebae *Acanthamoebae castellanii*, known to be a natural reservoir of *L. pneumophila*. Surfactin is a major class of cyclic lipopeptides abundantly produced by various *Bacillus* environmental isolates and remains the best known biosurfactant (Mora et al., 2015).

**FIGURE 5:**
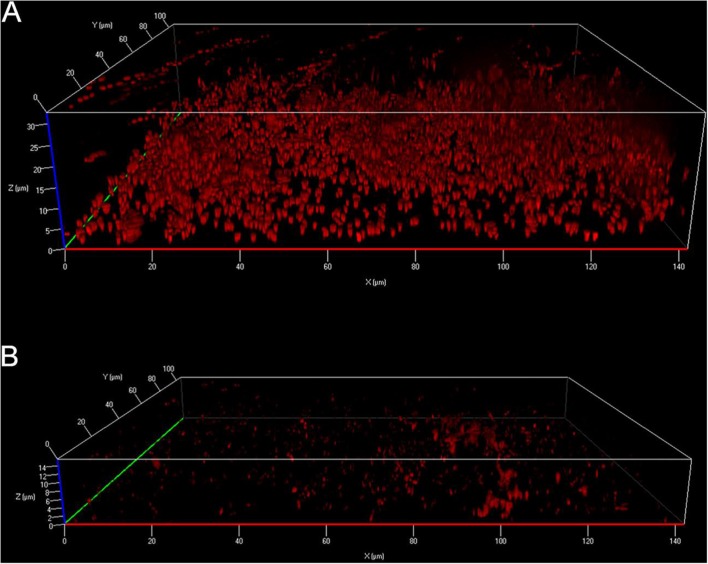
**Apotome imaging of surfactin-treated 6-day-old biofilms formed by *L. pneumophila*.** Biofilms were treated 2 h either with **(A)** ethanol as control or **(B)** 66 μg/ml surfactin (Source: Loiseau et al., 2015).

Lipopeptides, which constitute a specific class of microbial secondary metabolites, are well identified as antimicrobial agents (Ines and Dhouha, 2015). However, according to the literature on the antibacterial activities of purified or commercially purchased standard surfactins, few susceptible species have been reported (**Table 3**). It has to be noted that many studies did not separate surfactins from other produced lipopeptides or only tested the supernatant/crude extracts when performing antibacterial assays. So, the number of sensitive bacterial species is clearly underestimated. Surfactins were also found to breakdown *L. pneumophila* pre-formed biofilms but did not prevent biofilm attachment (**Figure 5**) (Loiseau et al., 2015) unlike biofilms produced by *Salmonella enterica* Serovar Typhimurium (Mireles et al., 2001).

**Table 3:** Minimum inhibitory concentrations (MIC) of purified or commercially purchased surfactins against selected bacterial strains.

Target bacteria	MIC	Producing strain	Antibacterial Assay	Reference
*E. coli* AS1.487	15.625 μg/ml	Commercially purchased	Microdilution	Huang et al., 2008
*P. syringae* pv *tomato* DC3000	25 μg/ml	Commercially purchased	Microdilution	Bais et al., 2004
*L. monocytogenes* 99/287RB6 strains	125 μg/ml 250 μg/ml 1 mg/ml	*B. subtilis* C4 *B. subtilis* G2III *B. subtilis* M1	*WDA	Sabate and Audisio, 2013
*S. enteritidis*	6.25 μg/ml	Commercially purchased	Microdilution	Huang et al., 2011
*V. anguillarum*	1.5 μg/ml	*B. amyloliquefaciens* M1	Not specified	Xu et al., 2014
*Legionella* sp.	1–4 μg/ml	Commercially purchased	Microdilution	Loiseau et al., 2015
*M. pulmonis* MpUR1.1	25.9 μg/ml	Commercially purchased	Microdilution	Fassi Fehri et al., 2007

*WDA: well diffusion assay.

Minimum inhibitory concentrations were determined against *L. pneumophila* Lens and for the formylated forms of the peptides except for the peptides indicated by *.

Concerning their mechanism of action, surfactins seem to act by direct lysis of negatively charged membranes (Buchoux et al., 2008). This lysis is driven by electrostatic repulsion between negatively charged acidic residues from surfactins and negative charges located on the lipid headgroups after penetration of the lipopeptide into the lipid bilayer, leading to complete destabilization of the planar membrane and small vesicles formation.

## Conclusion

*Legionella pneumophila* appears sensitive to various biomolecules including molecules that are poorly active against others bacteria like surfactin. However, it is important to keep in mind that *L. pneumophila* is not a routinely used bacterium when determining the antimicrobial potency of a given product in contrast to bacteria such as *E. coli*, *S. aureus*, or *P. aeruginosa*. Therefore, it is easy to understand why there are so little known anti-*Legionella* molecules available in the literature. On the other hand, the described compounds are very active against *L. pneumophila* compared to other bacteria. Does *L. pneumophila* possess some specificity that could explain this sensitivity? As all these compounds are membrane active, maybe a part of the answer is hidden in the composition of *L. pneumophila* cell envelope. The current knowledge about the structure and molecular composition of its cell envelope was recently reviewed, and authors highlighted several characteristics that deserve more attention (Shevchuk et al., 2011). Starting from the outside and proceeding inward, it appears that *L. pneumophila* LPS has a unique structure in comparison to the LPS of other Gram-negative bacteria. Due to high levels of long, branched fatty acids and elevated levels of *O*- and *N*-acetyl groups, this LPS is highly hydrophobic. The high level of PC is also striking as only 15% of all known bacteria have the ability to synthetize PC (Geiger et al., 2013; Sohlenkamp and Geiger, 2016). Nevertheless, the exact function of this phospholipid in bacterial cell envelopes remains unclear regarding the sensitivity of *Legionella* to antimicrobial compounds (Geiger et al., 2013). However, it has already been shown that activities of various biomolecules (AMPs, ApoLp-III) are modulated in presence of PC, even if the reason remains poorly understood. Moreover, the fatty acid composition of membranes also influences bactericidal properties. *L. pneumophila* possesses a high level of branched chain fatty acids, mainly in the stationary phase of growth (Verdon et al., 2011). This level was shown to be involved in the sensitivity of *L. pneumophila* to warnericin RK. Taken together, all those data highlighted several particularities of the envelope components already shown to be implicated in *Legionella* virulence (Shevchuk et al., 2011). Another striking feature is the high sensitivity of *L. pneumophila* to detergent (Verdon et al., 2009a), underlining the key role played by its membrane components. As all described active compounds target this cell compartment, establishing a link between sensitivity and membrane composition/arrangement is tempting. It appears that membrane thickness, fluidity, phospholipid composition and even presence of phospholipids clusters could be key parameters involved in *Legionella* sensitivity. Anyway, the lack of experimental data about mechanisms of action of active molecules is a bottleneck in the discussion. Further experimental investigations are clearly and rapidly needed to decipher the precise mechanisms of action of such biomolecules. However, the critical analysis of the literature presented here reveals that natural biomolecules could represent potent tools for the biological control of *L. pneumophila* and/or its natural hosts in water treatment industry, although additional experiments are needed to demonstrate how effective these antagonists would be under field conditions.

## Author Contributions

J-MB, SC, and JV conceived and designed the review. J-MB, SC, MS, EP, CL, WA, OL, and JV wrote the paper. JV coordinated the work.

## Conflict of Interest Statement

The authors declare that the research was conducted in the absence of any commercial or financial relationships that could be construed as a potential conflict of interest.

## Footnotes

1 http://www.dsmz.de/bactnom/bactname.htm

2 http://www3.epa.gov/
